# Mental health task-sharing in South Africa – a role for clinical associates?

**DOI:** 10.1186/s12913-022-08638-3

**Published:** 2022-10-08

**Authors:** Saiendhra Vasudevan Moodley, Jacqueline Wolvaardt, Christoffel Grobler

**Affiliations:** School of Health Systems and Public Health, Faculty of Health Sciences, University of Pretoria, Pretoria, 0084 South Africa

**Keywords:** Mental health, Task-sharing, Clinical associates, South Africa, Health workforce

## Abstract

**Background:**

South Africa (SA) lacks the specialised workforce needed to provide mental health services particularly in the public sector and in rural areas. Mid-level medical workers offer a potential option for mental health task-sharing in countries where they exist, including SA. The objectives of the study were to explore the roles that SA’s mid-level medical worker cadre (clinical associates) could play in mental health service delivery, and to explore views on advanced training in mental health for this cadre.

**Methods:**

This was an explorative, qualitative study involving key informants linked to the three clinical associate training programmes in SA. A total of 19 in-depth interviews were conducted with university-based academic staff, facility-based trainers, and student representatives. The interviews were audio-recorded and professionally transcribed. Thematic analysis was conducted with the aid of Atlas.ti software. Themes and subthemes were identified.

**Results:**

The first theme identified was ‘there is a place for them at the table’. Participants felt that there was a definite role for clinical associates in mental health service provision. The levels of care thought most appropriate were primary health care facilities and district hospitals. The most frequently identified role for clinical associates was in providing immediate care and stabilising mental health patients presenting in emergency settings. Other potential settings included inpatient wards, outpatients’ departments, and in communities (e.g. home visits). The second theme identified was ‘earning a seat at the table’. There was virtually unanimous support for additional training and in particular a postgraduate clinical specialisation in mental health. Participants felt a clinical specialisation in mental health would strengthen the health system by addressing workforce shortages as well as access and equity issues. They also held the view it would strengthen the profession by creating a career path and providing more employment opportunities for clinical associates.

**Conclusions:**

There was broad support for a role for clinical associates in mental health service delivery in SA as well as for the establishing a clinical specialisation in mental health for clinical associates. Clinical associates with advanced training in mental health could potentially play an important role in rural settings to alleviate the shortage of specialist mental health practitioners.

**Supplementary Information:**

The online version contains supplementary material available at 10.1186/s12913-022-08638-3.

## Background

There is evidence of severe shortages of the health workforce required for mental health in many low- and middle-income countries [1, 2]. Bruckner et al*.*[1] found that all low-income countries and the majority of middle-income countries in their sample did not have sufficient mental health professionals to provide a core set of interventions. The disparities in the distribution of the global mental health workforce is highlighted by the World Health Organization (WHO) African Region having a median number of 1.6 mental health workers per 100 000 population in 2020 compared to the WHO European Region which had a median of 44.8 per 100 000 population [3].

In 2017, South Africa (SA) had 1.52 psychiatrists per 100 000 population compared to a median number of psychiatrists per 100 000 population of 2.1 for upper-middle-income countries and 12.7 for high-income countries [4, 5]. There is a lack of data for other cadres of mental health workers. Wishnia et al*.*[6] project that psychiatry will be one of the specialties in SA with an extremely large deficit of specialists per 100 000 population by 2040 when compared to the SA’s National Department of Health target adjusted for epidemiology. There are also substantial disparities in the distribution of mental health workers between the private and public sectors of SA as well as urban and rural areas. In 2019, there were 0.38 psychiatrists per 100 000 population in SA’s public sector that serves the majority of its population compared to 4.98 per 100 000 population in the private sector [6]. In a situation analysis involving 160 public rural primary healthcare facilities (including district hospitals) in SA, only seven psychiatrists were employed in these settings (0.03 psychiatrists per 100 000 population) [7]. De Kock et al*.*[8] found that only 62 (38.7%) of 160 public rural primary healthcare facilities employed mental health nurses. A population of over 17 million was being served by a total of 116 mental nurses (0.68 mental health nurses per 100 000 population) [8].

The shortage of specialist mental health workers has necessitated the use of task shifting and task sharing approaches in many countries [2, 9, 10]. A shortage of psychiatrists prompted Sri Lanka to create a three-month psychiatry training course for medical officers who are then stationed at primary and secondary levels [2, 11]. Studies have shown that the use of non-specialist nurses to deliver mental health services can be beneficial [12–14]. An audit of depression case management by practice nurses in general practitioner settings in England found both clinical improvement in patients and patient satisfaction with the service [12]. The practice nurse intervention involved medication management, behavioural activation, symptom assessment and non-responder identification [12]. Clinical benefit was demonstrated for moderate to severe depression (but not for mild depression) in a cluster randomised trial in the United States of America (USA) where depression care management consisting of goal setting, education, medication management, symptom assessment, and care co-ordination was integrated into the routine practice of nurses doing home visits to Medicare patients 65 years and older [13]. There is evidence from a task-sharing intervention in Ethiopia that primary health care nurses can play an important role in the diagnosis and treatment of patients with severe mental illness [14]. Research from southern Africa suggests that lay health workers could play an important and effective role in mental health service delivery [15–18].

A number of countries have initiated mid-level medical workers programmes to address health workforce shortages [19]. Physician assistants have been trained in the USA and similar cadres are found in Australia, Canada, Netherlands and the United Kingdom [20]. Training of mid-level medical workers in sub-Saharan Africa started with clinical officers being trained in Kenya [21]. South Africa began training of mid-level medical workers named clinical associates with the first cohort entering the South African health system in 2011 [22]. Clinical associates complete a three-year undergraduate Bachelor of Clinical Medical Practice or Bachelor of Medical Clinical Practice degree [22]. Training takes place mainly at district hospitals and consists of case-based learning and early exposure to clinical work [22]. The scope of practice of clinical associates includes taking an history, performing an examination, performing diagnostic procedures, formulating a diagnosis, developing a management plan and performing specified procedures under supervision [23].

The degree of involvement of mid-level medical workers in the delivery of mental health services varies between countries where these cadres exist. While the undergraduate training programmes for clinical associates in SA does include mental health, there is no evidence to suggest they are widely used to deliver mental health services. The objectives of the study were to explore the roles clinical associates in SA could play in mental health service delivery, and to explore views on advanced training (including a clinical specialisation) for this cadre in mental health.

## Methods

### Study design

We used an explorative, qualitative approach comprising in-depth interviews. The in-depth interviews consisted of two components. The first component addressed the mental content of the clinical associate training programmes and those results are reported elsewhere [24] while the second component explored participants’ views on clinical associate roles and advanced training related to mental health.

### Study setting

This was a multisite study involving the three universities offering clinical associate undergraduate degrees in SA.

### Study participants

The study involved key informants linked to the three clinical associate training programmes. The key informants were university-based academic staff, facility-based trainers, and student representatives who were selected using purposive sampling. The target was to include a minimum of five individuals linked to each of the three programmes regardless of data saturation.

### Data collection

Participants were invited to participate in the in-depth interviews via e-mail and appointments for interviews with each participant were made once they agreed to participate. Blackstone [25] highlights the importance of sharing information to balance the power differential between interviewer and interviewee. In this study, the interviewer shared some details about his background, the reasons behind his interest in the topic, and gave the participants a clear idea of the intent of the research including sharing the rationale and research questions with them.

In-depth interviews were conducted using an interview guide that was developed for this study (supplementary file 1). The questions related to mental health in the curriculum, participant views on clinical associate roles in mental health service provision, and participant views on advanced training. Interviews were conducted using videoconferencing but were audio recorded only. Participants were given the option of using either Microsoft Teams or Zoom. Hand-written notes were also taken during the interview. Only the researcher conducting the interview and the participant were present during each of the interviews. The duration of interviews ranged from 28 to 58 min (including the first part of the interview which dealt with the curriculum). The recordings were transcribed professionally.

### Data analysis

An inductive data analysis approach based on constructivist grounded theory was utilised. ATLAS.ti software was used to aid the analysis. Colaizzi’s seven-step method for data analysis was used to guide the analysis process [26]. The first author read through the transcribed interviews, identified significant statements (which were coded), and formulated meanings [26]. The codes were then aggregated into categories [27] and clustered into themes [26]. The codes, categories and themes were reviewed and confirmed by the second author.

## Results

Of those that responded to the e-mail invitations that were sent out, 19 individuals agreed to participate while three individuals declined (one citing time constraints and two felt they were not appropriate based on their roles). In-depth interviews were conducted between 25 March 2021 and 29 July 2021 with all 19 individuals who agreed to participate. No repeat interviews were conducted.

All three universities were represented with seven participants from one university and six participants from the remaining two universities. The professional categories of the interviewees were clinical associates or foreign-trained equivalent (*n* = 11), family physicians (*n* = 4), medical officers (*n* = 3) and psychiatrists (*n* = 1). Three of the clinical associates were student representatives from the previous graduating class at each university. The sample consisted of 11 females and eight males.

The themes and subthemes that emerged from the in-depth interviews are shown in Table 1. The participants felt there was role for clinical associates in mental health and identified the settings and mental health tasks where they could play a role indicating ‘there is a place for them at the table’. However, their current training may not be adequate to fulfil their potential role in mental health and advanced training through a clinical specialisation and short courses could assist them in ‘earning a seat at the table’.

**Table 1:** Themes and Subthemes

Themes	Subthemes
There is a place for them at the table	Support for a clinical associate role in mental health
	Appropriate settings to render mental health services
	Potential mental health “tasks”
Earning a seat at the table	The need for a clinical specialisation in mental health
	The need for short courses in mental health

### Theme 1: There is a place for them at the table

#### Subtheme: Support for a clinical associate role in mental health

Participants could envisage potential roles and a range of potential health “tasks” for clinical associates in mental health predominately in the settings in which they currently work. There was widespread support amongst the participants for the utilisation of clinical associates in the provision of mental health services as they already have basic mental health training, consultation and communication skills. One participant noted that allowance was made for this in the Regulations[23] by allowing a supervising medical practitioner to delegate any task to a clinical associate who has the requisite training and experience. Participants expressed the view that clinical associates would add value in mental health service delivery:

“*…they are very relatable to a majority of people that are struggling with mental health issues…. And putting clinical associates at the front of these conversations and seeing these patients, is definitely a good thing. So, I don’t have a limit to where I think their role is. I think they could fit in anywhere in the South African landscape for mental health, anywhere where the help is needed.*” (clinical associate 07)

#### Subtheme: Appropriate settings to render mental health services

Participants envisaged the most appropriate settings for clinical associates to render mental health services as primary health care (PHC) facilities and district hospitals. The reasoning for district hospitals was that *“clinical associates have been trained in district hospitals, a foundation, a starting place for them is there*” (clinical associate 08) and that this is a reflection of the original intention: “…*we viewed clinical associates right from the time of inception, we viewed them as district hospital and down to PHC*.” (medical officer 01) Their existing training in mental health was viewed as a barrier to providing mental health services at other levels: “*I feel like those topics, those conditions are adequate for us to manage, especially specifically at the places that we are allocated, so which is mainly district hospitals and the primary healthcare system*.” (clinical associate 06).

Provision of mental health services at community level was mentioned as an issue that needed to be addressed. The potential value of involving clinical associates at this level was noted:

“…*one of the advantages is, clinical associates can write prescriptions and so at the community health centre level, depending on who’s staffing it, clinical associates could write prescriptions and then those patients could remain in the community and receive their medicines, as opposed to having to go to the district hospital or tertiary hospital*.” (clinical associate 09)

A few of the participants felt that clinical associates could play a role at regional, tertiary and even specialised psychiatric hospitals as well as in the private sector:

“…*the medical world is open to them given that they have the right training, given that they have the right supervision, given that they have the right mentorship to be trained up, and I don’t think there should be any limit to the location of where clinical associates could be placed or worked or that level of patient to work with. You know, if there’s a need for a clinician to help in mental health in the private area, if there’s a need to help at a tertiary hospital, if there’s a need at the district hospital, or at the CHC, that would be great.”* (clinical associate 09)

#### Subtheme: Potential mental health “tasks”

The most frequently identified role for clinical associates was in providing immediate care to mental health patients presenting in emergency settings. Specific examples provided by participants included the initial management and stabilisation of violent, psychotic and suicidal patients:

*“I feel I would be competent to do emergency mental health. Like if I’m in casualty and I have a violent patient or I have a patient with a suicidal attempt, or I have a patient that’s hallucinating or confused or they have alcohol or drug abuse kind of problems, I would be able to help them mostly.”* (clinical associate 04)

However, some participants expressed some misgivings regarding their readiness to take on such a role:

“…*the immediate acute care… is quite a difficult and a specialised field. So, on the one hand you would like as many people to be competent in doing that, on the other hand it’s not as simple as it seems. And so, I’m not sure if they are the right cadre to do that. And I think it will largely depend on what their role would be in terms of their scope of practice and their job descriptions*.” (psychiatrist 01)

Inpatient management of mental health patients was limited to a rudimentary level based on their current training. A potential role for inpatient care was to care for those who have been assessed rather than doing the initial assessment themselves. The possibility to play an expanded role in inpatient care was largely dependent on further training: *“And with those basic skills and a little bit more training for mental health, I think that incorporating a clinical associate in a psychiatry ward…it’s a given.”* (clinical associate 07) One role that they could perform after a period of supervision was the 72-h observation of patients admitted under South Africa’s Mental Health Care Act, 2002 as *“they can grow into that role quite easily, but their initial six months would need some supervision and input.”* (family physician 01).

Participants easily envisaged that clinical associates had a role to play in outpatient settings including outpatient departments at hospitals, community health centres, clinics, and in private practices. The roles included taking a history and doing the mental status examination, performing a suicide risk assessment, prescribing medication such as antidepressants and anxiolytics, providing supportive counselling, and following up patients. A participant noted that *“psychiatric medications should maybe be a topic to be discussed and explored with psychiatrists and the mental healthcare service, which ones would be appropriate for clinical associates to adjust and to initiate*.*”* (family physician 01) Clinical associates could play a role in counselling patients with depressive, anxiety and substance use disorders. There was hesitancy around clinical associates role in diagnosing and initiating treatment in contrast to the level of confidence regarding their role in managing further care:

“*I think for the diagnosing purposes…they should refer patient for diagnosis. Once the patient is diagnosed with the selected treatment they can manage based on guidance from the specialist, you know, like a down referral. They are able to follow the script; they are able to follow the advice. But when it comes to diagnosis and prescribing a treatment for the first time, I wouldn’t be so sure.*” (medical officer 03)

As part of the move towards community-orientated primary care in SA, it was also suggested that clinical associates could do mental health home visits which could include both initial assessment and follow up. The latter would include assessing adherence to medication. A few participants suggested that clinical associates could also be utilised in mental health promotion activities e.g. “*engaging in some sort of community work when it comes to mental health campaigns, or just to get information out there to families, communities, and just South Africa as a whole.”* (clinical associate 01) It was suggested they play a role in school mental health. Screening for alcohol and drug abuse as well as screening patients with chronic medical conditions for mental health issues were suggested as other roles they could play.

### Theme 2: Earning a seat at the table

#### Subtheme: The need for a clinical specialisation in mental health

While participants clearly felt there was a role from clinical associates in providing mental health services, they felt that there was a need for advanced training in the form of a clinical specialisation in mental health or short courses in mental health. There was substantive support for a clinical specialisation in mental health for clinical associates in the form of an Honours degree or postgraduate diploma:

“*I definitely think it’s something worthwhile initiating. There’s a need and clinical associates would be appropriate to learn and provide that service, definitely. So I’m very supportive of getting such a course going and getting clinical associates the appropriate regulatory approval to actually function as mental healthcare providers…”* (family physician 01)

In fact, mental health should be prioritised when considering possible future specialisation offerings and the current burden of disease:

“*Now there are a number of disciplines I would think about for specialisation, and psychiatry is one of the key ones. Like there are about four disciplines I would think about. And psychiatry, because of the mental health burden in South Africa, psychiatry is an area that we really need to channel clinical associates into.*” (medical officer 01)

The reasons provided for the need for a clinical specialisation could be grouped into three categories namely strengthening the health system, strengthening the profession, and individual reasons.

Equitable access to mental health services was provided as a rationale for a specialisation in mental health by a few participants. It was felt that this could lead to an expansion of mental health services to underserved setting particularly rural areas.The burden that the current situation places on patients was noted:

“…*why should a patient who has a mental healthcare issue, that uses* (a rural district) *Hospital, for all of their other issues, why should they have to go to a different facility just because they have a mental health illness? It’s difficult for the patients, and therefore our hospitals should be able to ensure that they have adequate human resources for them.”* (clinical associate 03)

The significant impact that a clinical specialisation could have in strengthening mental health services offered by district hospitals was highlighted:

“…*it would really help because that would actually initiate creating maybe units for psychiatric patients within the district hospitals. Especially if there’s someone who is going to be devoted into actually managing that, overseeing that section of that hospital.*” (clinical associate 06)

A number of participants voiced concern regarding the lack of a career path and postgraduate opportunities for clinical associates. This has led to much frustration with many clinical associates because they realise after a short period that there no avenues for them to progress. The lack of opportunities leads to many of them opting to pursue a medical degree. A specialisation will create opportunities and make them a more valued member of the health workforce.It was suggested that a specialisation would give them additional employment opportunities specifically at specialised psychiatric hospitals. A specialisation would allow “*the clinical associate to continue their personal growth, to improve the category, to improve the salary, to even the perception of the people around them.*” (family physician 03) It would also “*contribute to them having a niche, having a more solid identity within the system. So, it would contribute to using the clinical associates more.*” (medical officer 01).

The thought was that a specialisation in mental health would give clinical associates, who are interested in mental health, an avenue to pursue this further:

“…*when they are brand new students and they come on the programme, there are some that are really passionate about mental health. But I think that passion ends up being sort of put to the side because there is just not the room for them to grow that interest...”* (clinical associate 03)

#### Subtheme: The need for short courses in mental health

Participants suggested that short courses in mental health may help close some gaps particularly as the duration of training in mental health is limited at undergraduate level:

“..*no one knows everything after an undergrad, but where there’s continuous learning, or they get these opportunities to really get more information, it helps them become more competent and confident and then it’s going to increase basically the quality of their practice.*” (clinical associate 01)

Participants cited evidence regarding the effectiveness of short courses to galvanise service provision – for example the role of clinical associates in circumcisions after completion of a short course – that could be applied to mental health. Short courses may also increase the confidence of the employer:

*“ … maybe a few short courses…and you could present these certificates to your manager, they’d feel more comfortable, clinical associates, seeing mental healthcare patients in the institution.”* (clinical associate 08)

The caution was that short courses do not usually include a clinical component with patient interaction which impacts its utility:

“*You’ve got to be out there. You’ve got to be seeing patients…our greatest opportunity is during the undergraduate years.* ” (family physician 04)

## Discussion

Our study explored the potential role for clinical associates in South Africa in mental health service provision and the need for advanced training. We found that ‘there is a place for them at the table’ as there was clear support for a role for clinical associates in the provision of mental health services. The appropriate settings to render mental health services were felt to be at primary care and district levels. There is some evidence that similar cadres already play such a role in similar settings in other countries in Africa. Clinical officers in Kenya working in primary care assess, diagnose and treat patients with psychosis and refer complex cases [28]. Malawian clinical officers who have undergone further specialist training in psychiatry form part of District Mental Health Teams based at district hospitals [29]. According to Ethiopia’s National Mental Health Strategy, health officers have an important role to play in mental health service provision at health centres together with general practitioners and nurses [30].

Potential mental health ‘tasks’ were identified that could be carried out by clinical associates. Their potential role in emergency mental health was highlighted and a broader role encompassing inpatient and outpatient care was considered possible provided that there was appropriate training, experience and supervision. The physician assistant programme in the USA may provide an indication of potential areas for mental health task-sharing. In 2019, 1.6% of physician assistants in clinical practice identified psychiatry as their principal clinical position [31]. They work in outpatient and inpatient settings [32] and their responsibilities include mental health screening, conducting the initial psychiatric assessment, diagnosis, psychiatric medication management, provision of education, counselling and psychotherapy, managing substance detoxification and rehabilitation, and managing psychiatric emergencies [33].

The evidence for effectiveness of similar mid-level medical worker cadres (such as physician assistants, clinical officers, physician associates) in providing mental health services in other countries is limited due to the lack of research in this area. McCutchen et al*.* [34] found that including a physician assistant in an assertive community treatment team improved access to preventative health services and primary care, access to psychiatric care, quality of psychiatric care, and referral processes. A study in Ethiopia evaluated an intervention in which trained primary care workers (health officers and nurses) assessed referrals of possible severe mental illness, made a diagnosis, and initiated treatment using the WHO mhGAP Intervention Guide [14]. The primary care workers adhered to recommended medication dose limits in all cases but only 29.8% of patients received minimally adequate treatment [14]. The intervention resulted in statistically significant improvements in symptom severity and disability [12]. A study in Kenya found it was feasible for clinical officers (and nurses) to implement the WHO mhGAP Intervention Guide and they can do so effectively as demonstrated by clinical, disability and quality of life outcomes [35].

The wider use of clinical associates in mental health service delivery could have a number of benefits including addressing health workforce shortages in underserved areas. The majority of clinical associates work in the public sector in SA [36]. There is evidence from the University of Pretoria across nine cohorts of clinical associate students that the majority of their clinical associate students intend working in rural areas [37]. Based on the experience within one Trust in the UK, Gill et al*.*[38] highlight a number of potential benefits of employing the UK’s mid-level medical worker cadre (physician associates) in mental health settings including addressing health workforce constraints as they can take on many of the responsibilities of junior doctors, ensuring continuity of care as they stay in posts longer then junior doctors, and their ability to work well within multidisciplinary teams.

We explored the support and rationale for advanced training in mental health to be offered to clinical associates. Advanced clinical training in mental health in the form of an Honour’s degree or postgraduate diploma was widely and enthusiastically supported. Such specialised mental health training already exists for similar cadres in a few African countries. In Malawi for example, clinical officers can pursue a two-year Bachelor of Science in Clinical Medicine (Mental Health) degree after their initial three-year diploma and one year of internship [29, 39]. A clinical officer who completes this programme is able to practise as a specialist psychiatric clinical officer [29]. Participants felt that there was also a place for short courses in mental health for clinical associates who might not necessarily want to pursue a specialisation in mental health. A clinical specialisation or short courses in mental health provides a means of strengthening their potential role in mental health service provision and thereby ‘earning a seat at the table’.

An Honours degree for clinical associates already exists for one discipline in SA viz. emergency medicine and mental health is a clear contender for the next discipline based on the prevalence of common mental disorders in South Africa, the burden of disease due to mental disorders, and the impact of COVID-19 on mental health [40–43]. Critically, clinical associates specialised in mental health could help address access and equity issues related to the maldistribution of specialist mental health practitioners between urban and rural areas, and public and private sectors [6–8]. A specialisation in mental health could also address the parallel need for a career path for clinical associates. Based on a study at the University of Pretoria, more than 80% of clinical associates planned on further studies after their undergraduate degree with substantial interest in pursuing a clinical specialisation or a medical degree [37]. The current limited opportunities to study further will encourage some to pursue medicine.

Given the findings of this study, future mental health workforce policy in South Africa needs to consider the utilisation of clinical associates in the delivery of mental health services. In districts that already employ clinical associates, additional in-service training should be offered to allow for mental health task sharing. The three universities offering clinical associate training programmes should consider the introduction of an advanced qualification in mental health for clinical associates. This should be done in consultation with SA’s National Department of Health to ensure appropriate posts are created. There are a number of possibilities for future research. We did not attempt to look in any detail at a potential model for advanced training in mental health which should be the subject of future research. Clinical associates should be included in research focussing on the effectiveness of mental health task sharing interventions.

### Limitations

This was an exploratory study of the views of participants involved in clinical associate training programmes in the country. The views of other stakeholders such as government were not elicited. Participants in this study are likely to be more favourably disposed than health workers in general to a role for clinical associates in mental health and a clinical specialisation given their professional backgrounds and their desire to create opportunities for clinical associates. As no pilot study was conducted, the initial interviews were used to guide prompts in the subsequent interviews. Participants were not as optimally involved post interview as they could have been as transcripts were not returned them for corrections and comments and they were not asked to provide feedback on the findings [44]. However, the manuscript preprint was shared with them as soon as it become available online.

## Conclusion

There was broad support for a role for clinical associates in mental health service delivery in SA particularly at primary and district levels and an expressed need that they need additional training in the form of short courses or a clinical specialisation in mental health. The clinical specialisation was deemed to have an additional benefit in terms of career progression for clinical associates. Future opportunities for advanced training in mental health for clinical associates could potentially alleviate the shortage of specialist mental health practitioners in rural settings, reduce the psychiatric workloads of non-specialist medical practitioners, and improve the quality of mental health care.

## Supplementary Information

**Additional file 1.** Interview Guide for In-depth Interviews.

## Data Availability

The dataset used and analysed for this study are available from the corresponding author on reasonable request.

## Abbreviations

SA: South Africa
UK: United Kingdom
USA: United States of America
WHO: World Health Organization
