# Supplementary material for: Mental health task-sharing in South Africa – a role for clinical associates?
Source: BMC Health Serv Res. 2022 Oct 8;22:1242. doi: 10.1186/s12913-022-08638-3 (PMC9547675; doi:10.1186/s12913-022-08638-3)
Supplement: Supplementary file 1 — Additional file 1. Interview Guide for In-depth Interviews. [file 12913_2022_8638_MOESM1_ESM.pdf]

Interview Guide for In-depth Interviews

Date:

Time of Interview:

University: UP / WSU / Wits

Interviewer:

Interviewee:

Position of interviewee:

Questions

1. What aspects of mental health/psychiatry are currently covered in the curriculum?

2. How is the mental health component delivered through formal teaching?

3. What practical training is given in mental health?

3a. Can you tell me about your psychiatry rotation if there is one?

4. What are your views on whether mental health is adequately covered in your curriculum?

4a. What are the gaps in mental health teaching (if any)?

5. Based on their existing training, what sort of mental health work do you feel clinical associates will be competent to do when they graduate?

6. What role(s) would you like to see clinical associates playing in mental health service provision both now and in the future?

7. What are you views on a clinical specialisation in psychiatry for clinical associates?
